# Kinetics of cytochrome P450 3A4 inhibition by heterocyclic drugs defines a general sequential multistep binding process

**DOI:** 10.1074/jbc.RA120.016855

**Published:** 2020-12-25

**Authors:** F. Peter Guengerich, Kevin D. McCarty, Jesse G. Chapman

**Affiliations:** Department of Biochemistry, Vanderbilt University School of Medicine, Nashville, Tennessee, USA

**Keywords:** cytochrome P450, enzyme kinetics, pre–steady-state kinetics, enzyme mechanism, enzyme inhibitor, UV–visible spectroscopy, 7-OBz, 7-benzoyl, CYP and P450, cytochrome P450, EI, enzyme inhibitor, OH, hydroxyl, pmol, picomole, POR, NADPH–P450 reductase, SVD, singular value decomposition, UPLC, ultraperformance liquid chromatography

## Abstract

Cytochrome P450 (P450) 3A4 is the enzyme most involved in the metabolism of drugs and can also oxidize numerous steroids. This enzyme is also involved in one-half of pharmacokinetic drug–drug interactions, but details of the exact mechanisms of P450 3A4 inhibition are still unclear in many cases. Ketoconazole, clotrimazole, ritonavir, indinavir, and itraconazole are strong inhibitors; analysis of the kinetics of reversal of inhibition with the model substrate 7-benzoyl quinoline showed lag phases in several cases, consistent with multiple structures of P450 3A4 inhibitor complexes. Lags in the onset of inhibition were observed when inhibitors were added to P450 3A4 in 7-benzoyl quinoline *O*-debenzylation reactions, and similar patterns were observed for inhibition of testosterone 6β-hydroxylation by ritonavir and indinavir. Upon mixing with inhibitors, P450 3A4 showed rapid binding as judged by a spectral shift with at least partial high-spin iron character, followed by a slower conversion to a low-spin iron–nitrogen complex. The changes were best described by two intermediate complexes, one being a partial high-spin form and the second another intermediate, with half-lives of seconds. The kinetics could be modeled in a system involving initial loose binding of inhibitor, followed by a slow step leading to a tighter complex on a multisecond time scale. Although some more complex possibilities cannot be dismissed, these results describe a system in which conformationally distinct forms of P450 3A4 bind inhibitors rapidly and two distinct P450–inhibitor complexes exist en route to the final enzyme–inhibitor complex with full inhibitory activity.

Cytochrome P450 (P450, CYP) enzymes are found throughout nature, from many bacteria to humans, and a recent UniProtKB search yielded >400,000 genes (www.uniprot.org/uniprot/?query=P450&sort=score). P450s are the major catalysts involved in the oxidation of steroids, terpenes, alkaloids, drugs, fat-soluble vitamins, pesticides, industrial chemicals, and chemical carcinogens (1, 2). Of the 57 human P450 enzymes, P450 3A4 is the most abundant, being located in liver and small intestine. Originally characterized as the enzyme involved in the oxidation of the antihypertensive drug nifedipine (3), this single enzyme has been shown to be the main one involved in the metabolism of 25 to 50% of marketed drugs (4, 5, 6). Its prominence among the P450s can be rationalized in the context of its high expression levels (5, 7, 8) and its large and malleable active site (9, 10, 11).

The prominent role of P450 3A4 in drug metabolism makes it a site of binding of inhibitors as well as substrates, and P450 3A4 is considered to be a major locus for problems with drug–drug interactions (12). A recent analysis reported that of ∼150 drugs approved by the US Food and Drug Administration between 2013 and 2017, approximately 65% were P450 3A4 substrates, 30% were inhibitors, and 5% were inducers of the enzyme (6). Adverse interactions are a major medical issue and responsible for many hospitalizations and deaths (13).

P450s, including P450 3A4, are subject to the same types of inhibition that enzymes generally are, both reversible and irreversible (14, 15). Reversible inhibition can be competitive, noncompetitive, uncompetitive (although a good example for P450 is missing), or “mixed” (a rather nonmechanistic term). Irreversible inhibition of P450s is generally mechanism based or suicidal, in which either the protein or heme is modified (16). Quasi-irreversible inhibition for P450s involves oxidation to a carbene or C-nitroso group that binds tightly to the ferrous form of the iron (17, 18). However, even with inhibitors that have been extensively studied (*e.g.*, ketoconazole and ritonavir), there is considerable variability of reported inhibition constants (19) and controversy as to the mechanism of inhibition (6, 20, 21, 22, 23, 24, 25, 26, 27).

The binding of a ligand to a P450 often (but not always) involves a change in the UV–visible absorbance spectrum, usually observed in the Soret band. A shift of the iron from a resting low-spin state to high-spin state, associated with at least a partial loss of the H_2_O ligand to the iron, is termed type I change (λ_max_ ∼390 nm). Type II change involves the formation of a low-spin iron bound to a nitrogen atom of a ligand (λ_max_ ∼430 nm) (28). These changes can be used to characterize the binding affinity of P450 and ligands. Alternate modes of binding, *e.g.*, with an H_2_O molecule “sandwiched” between the iron atom and a ligand, have also been described (29). P450 3A4 has been somewhat problematic in terms of studying ligand binding, in that kinetic (30, 31), spectral (32), and structural (11) evidence for multiple occupancy by ligands have been presented.

Although the binding of ligands to P450s had generally been considered to be rapid, we reported that the binding of both substrates (33) and inhibitors (34) was a multiphasic process and consisted of multiple steps, a phenomenon confirmed by Sevrioukova and Poulos (26, 35, 36). The mechanism has been interpreted in terms of a multistep process in which binding first occurs at a peripheral site (10) and then the ligand is delivered to the area near the heme iron, where the spectral change is manifested (33, 34, 36). Alternate proposals for multistep binding of azoles have also been presented (37).

Although the kinetics of the spectral changes of inhibitor binding could be modeled in terms of three steps (34), there are deficiencies in the system. We subsequently showed that substrate binding to P450 3A4 is dominated by a “conformational selection” model, as opposed to induced fit (38), as in the case of P450 17A1 (39). The results indicate that multiple species of P450 3A4 are in equilibrium in the absence of ligand, and one (or more) of these conformations then binds the ligand. The previously described model did not contain this element (33, 34). Recent work with human P450 17A1 and two azole-based inhibitors showed the existence of a series of intermediate spectra on the pathway to the final type II complex, including an initial complex with some type I high-spin iron character (40). The conclusion from the latter study was that the enzyme inhibition was associated with the initial complex formed with the inhibitor. However, the system was not analyzed using pre–steady-state kinetics of product formation, and Cheong *et al*. (41) have interpreted the results of binding of a different pyridine-based inhibitor (abiraterone) in the context of the phenomenon known as slow and tight-binding inhibition.

The nature of the binding of five known (and clinically relevant) inhibitors to P450 3A4 (Fig. 1) was further characterized using kinetic and spectral approaches. The results are interpreted in terms of a multistate reversible process in which the strongest inhibition is associated with the final type II low-spin complex. The kinetics of the process may be relevant to the phenomenon of time-dependent inhibition seen with P450 3A4 and numerous other drugs (20).

**Figure 1:**
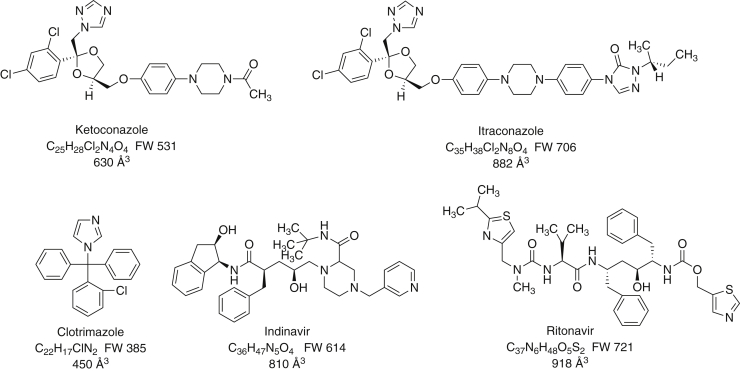
**Structures of inhibitors used in this work.** The formula weights and approximate molecular volumes (∼18 Å^3^/non-H atom) are also shown.

## Results

### Binding of 7-OBz quinoline to P450 3A4 and *O*-debenzylation

The addition of 7-benzoyl (7-OBz) quinoline to P450 3A4 induced type I difference spectrum (Fig. S1*A*), indicative of a low- to-high spin conversion of the iron atom (28). A titration yielded a *K*_d_ of 90 μM (Fig. S1*B*).

In contrast to several other P450 3A4 substrates (33, 38), the binding of 7-OBz quinoline was rapid, with >90% of the change finished in the first 200 ms (Fig. S1*C*). The estimated (single exponential) *k*_obs_ was 27 ± 1 s^−1^ at a 7-OBz quinoline concentration of 62 μM.

The *K*_m_ for 7-OBz *O*-demethylation in a reconstituted P450 3A4 system (42, 43) was 21 ± 3 μM, and the *k*_cat_ was 58 min^−1^ (Fig. S2).

### Inhibition of P450 3A4

**Figure 2:**
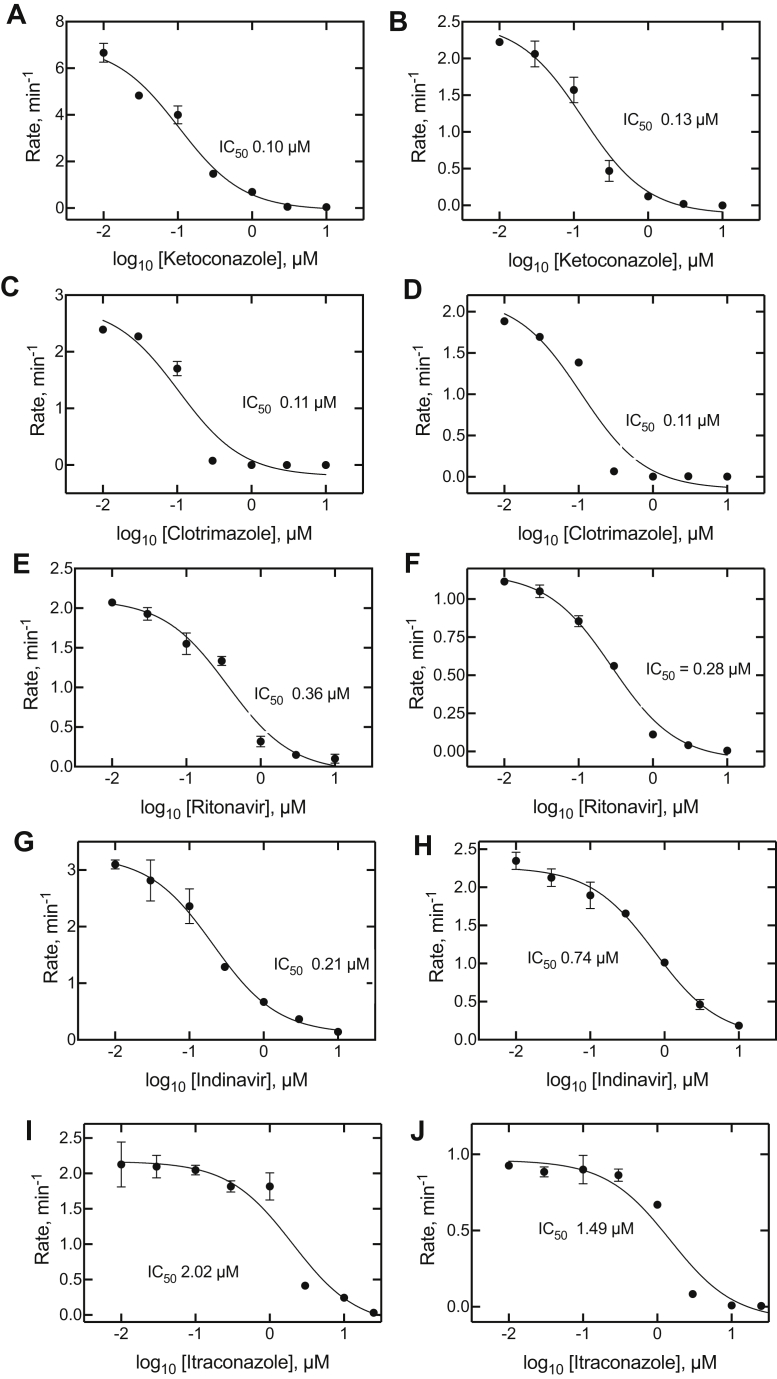
**Inhibition of cytochrome P450 3A4-catalyzed 7-benzoyl quinoline *O*-debenzylation and testosterone 6β-hydroxylation.** The steady-state rates are plotted as functions of (log_10_) inhibitor concentration, with the inhibitor indicated on the *x*-axis labels. *A*, *C*, *E*, *G*, and *I* (left panels): 7-benzoyl quinoline *O*-debenzylation; *B*, *D*, *F*, *H*, and *J* (right panels): testosterone 6β-hydroxylation. IC_50_ values are shown on the graphs. All fits had *r*^2^ values of ≥0.94. The 95% confidence intervals were as follows: *A*, 0.07 to 0.17 μM; *B*, 0.08 to 0.22 μM; *C*, 0.05 to 0.23 μM; *D*, 0.05 to 0.24 μM; *E*, 0.22 to 0.60 μM; *F*, 0.22 to 0.36 μM; *G*, 0.14 to 0.31 μM; *H*, 0.52 to 1.07 μM; *I*, 1.1 to 3.8 μM; and *J*, 0.94 to 2.7 μM.

The five inhibitors (Fig. 1) were tested for their ability to inhibit the testosterone 6β-hydroxylation activity of P450 3A4 (Fig. 2). As expected, all were highly effective. All five were also potent inhibitors of P450 3A4–catalyzed 7-OBz quinoline *O*-debenzylation activity. The IC_50_ values were submicromolar in all cases except for itraconazole, which presented technical problems because of solubility (see later). However, the low micromolar IC_50_ values for testosterone 6β-hydroxylation are consistent with the 7-OBz quinoline inhibition results. Even though there is some evidence for differences in the P450 reactions with the two substrates (44), the general similarity of the IC_50_ values for the two reactions suggests that the use of the continuous fluorescence assay with 7-OBz quinoline is valid as a surrogate for testosterone 6β-hydroxylation.

### Kinetics of recovery of P450 3A4–inhibitor complexes

If an enzyme is inactivated by a mechanism-based inhibitor, the inactivation is generally irreversible and cannot be rescued by the addition of substrates (45). However, in slow tight-binding inhibition, the conformation of the inhibitor-bound enzyme is in equilibrium with a form in which inhibitor can be released and replaced with substrate (46).

**Figure 3:**
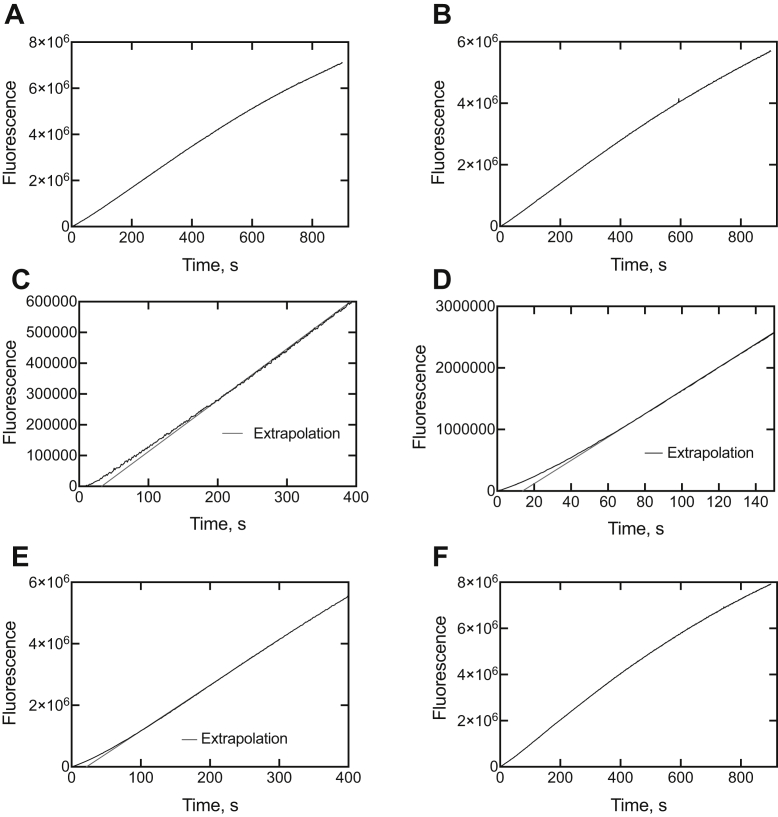
**Kinetics of recovery of catalytic activity from cytochrome P450 3A4–inhibitor complexes upon addition of 7-benzoyl quinoline.***A*, control (no inhibitor); *B*, ketoconazole; *C*, clotrimazole; *D*, ritonavir; *E*, indinavir; and *F*, itraconazole. Linear extrapolations to zero product formation are shown (*red lines*) in parts *C*, *D*, and *E*.

P450 3A4, in the presence of an equimolar concentration of each inhibitor (Fig. 1), was mixed with an NADPH-generating system, and then the substrate 7-OBz quinoline was added to initiate the *O*-debenzylation reaction. Extrapolation of the linear phases of the data showed lag phases of ∼45, 15, and 25 s for clotrimazole, ritonavir, and indinavir, respectively (Fig. 3).

The *K*_d_ for 7-OBz quinoline (90 μM, see previously) and its rate of conversion to (the fluorescent) product was used in KinTek Explorer (KinTek) modeling, but the output showed that the lags could be explained by a two-species and very tight-binding mechanism (results not presented), and alternate strategies to elucidating the kinetic mechanism were considered.

### Pre–steady-state kinetics of P450 3A4 inhibition

**Figure 4:**
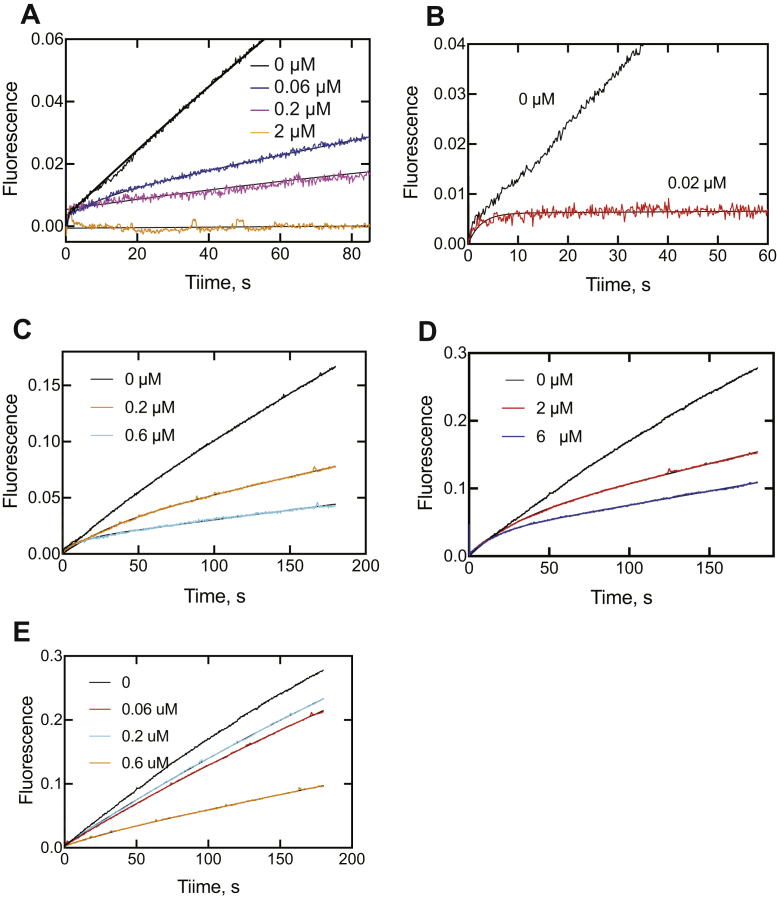
**Pre–steady-state kinetics of inhibition of cytochrome P450 3A4-catalyzed *O*-debenzylation of 7-benzoyl quinoline.** Plots of Δ*F*_410/>510_ are shown for *A*, ketoconazole; *B*, clotrimazole; *C*, ritonavir; *D*, indinavir; and *E*, itraconazole. The (final) inhibitor concentrations are shown on the individual graphs. The data were fit to the equation *y* = *A*(1 − e^−*k*^_1_^t^) + *k*_ss_t (Table 1).

In an alternate approach to the analysis of P450 3A4 inhibition, 7-OBz quinoline reactions were initiated by the simultaneous addition of *both* NADPH and an inhibitor to a reconstituted P450 3A4 system, using a stopped-flow fluorimeter (Fig. 4), to allow for pre–steady-state kinetic analysis. The initial binding of the inhibitors is rapid (see later), and there was no detectable lag for P450 reduction by NADPH–P450 reductase under these conditions, as indicated in the traces without inhibitor. These experiments are sensitive to the concentration of inhibitor, in that useful kinetic data cannot be obtained when inhibition is either too weak or too strong, in that the curvature is important in analysis of the plots (40). This kinetic approach also relies on the use of many data points, and a continuous trace of product formation is ideal.

A curvilinear approach to steady-state inhibition was not apparent in the case of itraconazole, but curvature was observed with the four other inhibitors at the indicated concentrations (Fig. 4). For instance, with ketoconazole (Fig. 4*A*), the *r*^2^ value was 0.99 for the line obtained with the uninhibited reaction but only 0.92 and 0.91 with the ketoconazole concentrations of 0.06 and 0.2 μM, respectively. The plots were analyzed by a combination log-linear equation of the form *y* = *A*_0_(1 − e^−*k*^_1_^t^) + *k*_ss_t, where *k*_1_ is the exponential rate of approach to the steady state and *k*_ss_ is the steady-state rate. The estimated rates and half-lives are listed in Table 1. Although the fitting is reminiscent of burst kinetic behavior (*i.e.*, in which a rate-limiting step follows product formation [15, 47]), the situation here is the exponential approach of an enzyme–inhibitor (EI) complex to the fully inhibitory form (15).

**Table 1:** Comparison of rates of interaction of inhibitors with P450 3A4

Inhibitor	Onset of 7-OBz quinoline *O*-debenzylation inhibition^a^			Spectral binding (SVD)^b^		
	Concentration, μM	Rate (*k*_1_), s^−1^^a^	*t*_1/2_, s	Concentration, μM	*k*_1_, s	*k*_2_, s^−1^
Ketoconazole	0.06	0.14 ± 0.01	4.8	15	0.38 ± 0.06	0.32 ±0.07
	0.2	0.87 ± 0.21	0.8			
Clotrimazole	0.02	0.39 ± 0.03	1.8	15	0.092 ± 0.01	0.016 ± 0.005
Ritonavir	0.02	0.028 ± 0.001	24	15	0.34 ± 0.10	0.32 ± 0.10
	0.06	0.090 ± 0.003	7.8			
Indinavir	2	0.034 ± 0.001	20	15	0.73 ± 0.10	0.61 ± 0.08
	6	0.067 ± 0.001	10			
Itraconazole				15	0.30 ± 0.10	0.61 ± 0.08

7-OBz, 7-benzoyl; P450, cytochrome P450; SVD, singular value decomposition.

a Rates are from the traces in Figure 4 fit to the equation *y* = *A*(1 − e^−*k*^_1_^t^) + *k*_ss_t (set in GraphPad Prism as *Y* = *A*(1 − exp[−Kfast ∗ *X*] + [Kslow ∗ *X*])) (78, 79). *t*_1/2_ = 0.693/*k*_1_.

b From the SVD analyses in Figures 9 and S11–S14.

**Figure 5:**
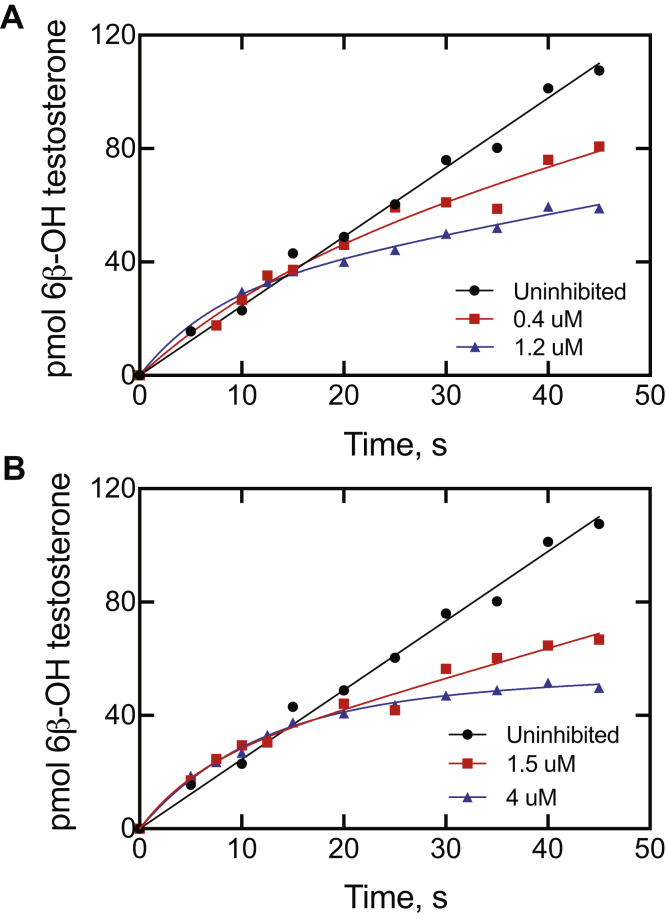
**Pre–steady-state kinetics of inhibition of cytochome P450 3A4-catalyzed 6β-hydroxylation of testosterone.** Plots of product formation are shown: *A*, no inhibitor added (●), 0.4 μM ritonavir (▪), and 1.2 μM ritonavir (▲). *B*, no inhibitor added (●, same plot as in part *A*), 1.5 μM indinavir (▪), and 4 μM ritonavir (▲). The data points for the uninhibited reaction were fit by linear regression. The data points for the inhibitors in parts *A* and *B* could be fit to the expression *y* = A (1 − e^−*k*^_1_^t^) + *k*_ss_t: *A*, *k*_1_ = 0.062 s^−1^ (*t*_1/2_ 11 s) and *k*_ss_ = 0.086^−1^ (28% of uninhibited rate) for 1.2 μM ritonavir; *B*, *k*_1_ = 0.17 s^−1^ (*t*_1/2_ 4.0 s) and *k*_ss_ = 0.13 s^−1^ (42% of uninhibited rate) for 1.2 μM ritonavir.

Similar studies were done with a well-established marker reaction of P450 3A4, testosterone 6β-hydroxylation (3), and the inhibitors ritonavir and indinavir (Fig. 5). These results are more difficult to analyze in that the assays are not continuous, as in the case of the fluorescence assays with 7-OBz quinoline (Fig. 4). The curvilinear plots are qualitatively very similar to those seen in the inhibition of 7-OBz quinoline *O*-debenzylation (Fig. 4), in which the development of inhibition is not immediate and the *t*_1/2_ for the approach to the steady-state level of inhibition is on the order of seconds.

## Spectral changes associated with binding of inhibitors to P450 3A4

### Preliminary experiments

**Figure 6:**
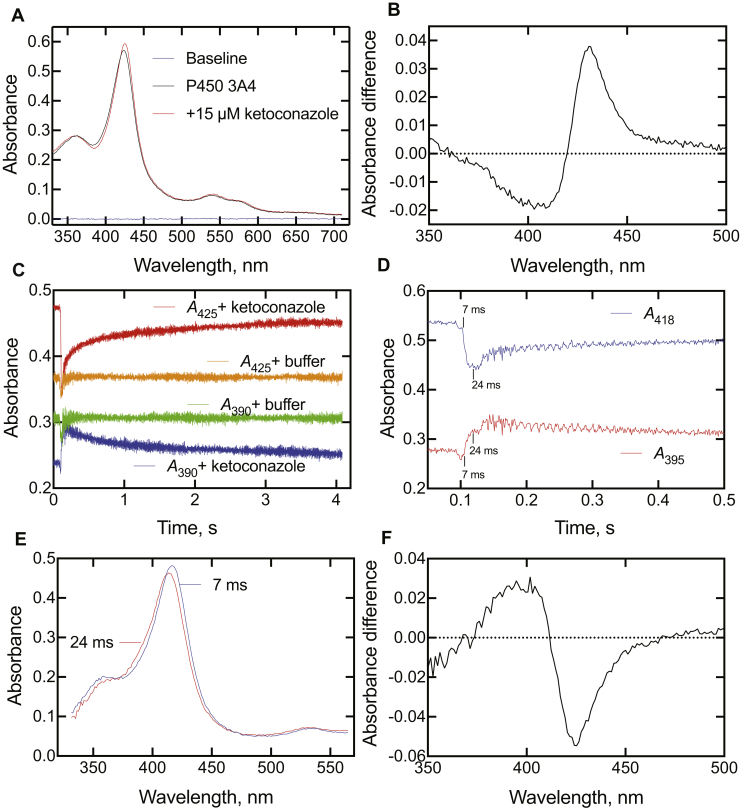
**Absorbance changes at 390 and 425 nm observed upon mixing ketoconazole with cytochome P450s (P450s).***A*, steady-state spectra of P450 3A4 (5 μM) in the absence and presence of 15 μM ketoconazole (in 100 mM potassium phosphate, pH 7.4). Addition of more ketoconazole did not produce further changes, as would be expected from the submicrometer IC_50_ (Fig. 2) and *K*_d_ values for ketoconazole and P450 3A4. *B*, difference spectrum from part *A*, with the P450-only spectrum subtracted from the spectrum obtained in the presence of ketoconazole. *C*, P450 3A4 (2 μM, final) was mixed with ketoconazole (15 μM). The indicated traces were obtained when the P450s were mixed with only the buffer (100 mM potassium phosphate, pH 7.4). The data were collected in the OLIS Show Pre-trigger Mode, with 0.1 s of data from the previous run (completed reaction) shown prior to actual mixing. *D*, expansion of data from experiment in part *C*, with the reaction being observed after 0.1 s (100 ms). The time points (7 and 24 ms) are calibrated for time after the initiation of the reaction (100 ms). *E*, spectra obtained upon P450 3A4 and ketoconazole 7 and 24 ms after reaction (from parts *C* and *D*), with the 7 ms spectrum reflecting mostly unbound P450 and the 24 ms spectrum reflecting the first observed P450·ketoconazole complex. *F*, difference spectrum generated from part *E* by subtracting the 7 ms spectrum from the 24 ms spectrum.

Ketoconazole has long been known to be a type II P450 ligand (binding of a nitrogen atom to the heme iron) (26, 34, 48, 49), and the spectral changes because of steady-state binding to P450 3A4 are shown in Figure 6*A*. Although the overall change in the spectra may seem small, subtraction yields a classic type II difference spectrum (Fig. 6*B*). P450 3A4 (2 μM) was mixed with 15 μM ketoconazole (final concentrations in cell), and spectra were recorded every 1 ms. The absorbance at 390 nm showed a rapid increase, followed by a slower multiphasic decrease (Fig. 6*C*). The trace at 425 nm was in the opposite directions, with a decrease followed by an increase to the final endpoint. (It should be pointed out that data were collected in the OLIS “Show Pre-trigger Mode,” with a short amount of data from the previous run (completed reaction) shown prior to mixing; this approach is useful in that the expected endpoint is displayed and provides a visual estimate of whether the reaction has gone to completion in the time frame of the reaction being monitored.) No changes were observed when only the buffer (100 mM potassium phosphate, pH 7.4) was mixed with enzyme (Fig. 6*C*), aside from the mixing artifact (refractive index change). Expansion of the early time point region following mixing showed the kinetics of changes at two wavelengths (395 and 418 nm), which appear to be exponential in generating a new complex (Fig. 6*D*). On the basis of the profiles in Figure 6*D*, the data at 7 ms (after the “trigger”) and 24 ms were selected as representing predominantly the uncomplexed P450 3A4 (7 ms) and the first P450·ketoconazole complex (24 ms) and were used to generate the corresponding spectra at those time points (Fig. 6*E*). The blue-shifted (hypsochromic) shift is evident. The change is further evidenced in type I difference spectrum generated by subtracting the 7 ms spectrum from the 24 ms spectrum (Fig. 6*F*). In subsequent spectral presentations (see later), the spectra at times of 32 to 96 ms after mixing (trigger) are presented as largely being this initial blue-shifted complex.

### Effect of cytochrome b_5_

**Figure 7:**
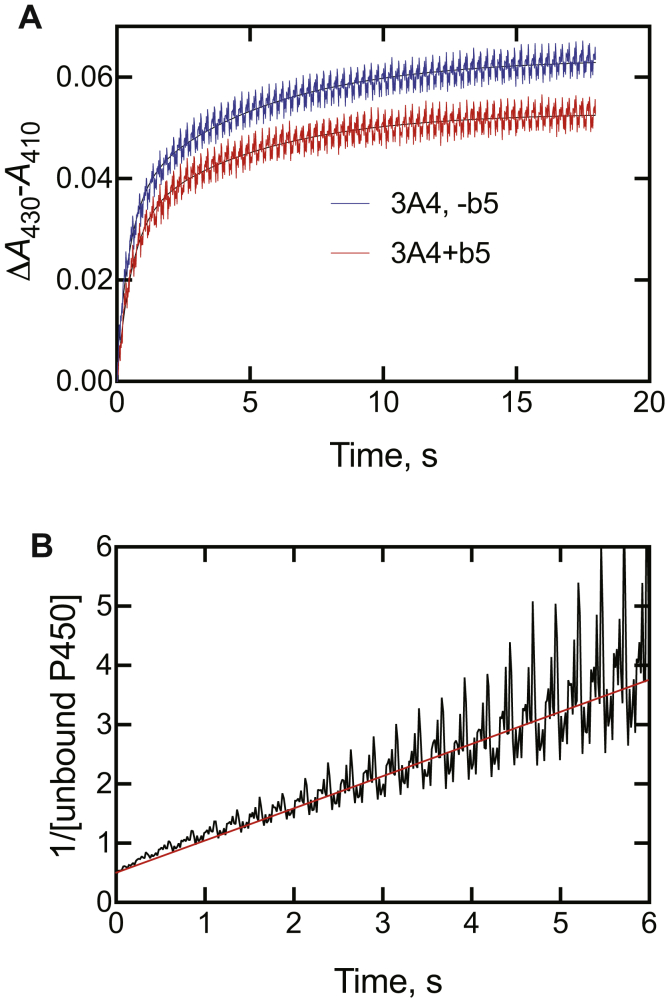
**Effect of cytochrome *b***_**5**_**on spectral interaction of cytochome P450s (P450s) with ketoconazole.** P450 (2 μM, final) was mixed with ketoconazole in the absence or presence of 2 μM cytochrome *b*_5_. *A*, P450 3A4 (±cytochrome *b*_5_) mixed with 2 μM ketoconazole. *B*, second-order plot of binding data (from part *A*, the absence of cytochrome *b*_5_). With equal amounts of P450 3A4 and ketoconazole, the reaction *X* + *Y* → *Z* (where *X* is P450 3A4, *Y* is ketoconazole, and *Z* is the complex) is mathematically equivalent to 2*X* → *Z* and a plot of 1/(unbound P450 [X]) versus time yields an apparent second-order rate constant as the slope (5 × 10^5^ M^−1^ s^−1^) (52).

Some of the reactions catalyzed by P450 3A4 are stimulated by cytochrome *b*_5_ (3, 50, 51). The presence of cytochrome *b*_5_ (equimolar) had only a small effect on the amplitude of the reaction of ketoconazole (2 μM final concentration) with P450 3A4 (2 μM) (Fig. 7*A*).

With equal amounts of P450 3A4 and ketoconazole, the reaction *X* + *Y* → *Z* (where *X* is P450 3A4, *Y* is ketoconazole, and *Z* is the complex) is mathematically equivalent to 2*X* → *Z*, and a plot of 1/[unbound P450] versus time (Fig. 7*B*) yields an apparent second-order rate constant as the slope (52). However, this value was low (5.4 × 10^5^ M^−1^ s^−1^) and is deficient in that the system is more complex than a simple two-state reaction (see later).

### Kinetics of binding of inhibitors to P450 3A4

**Figure 8:**
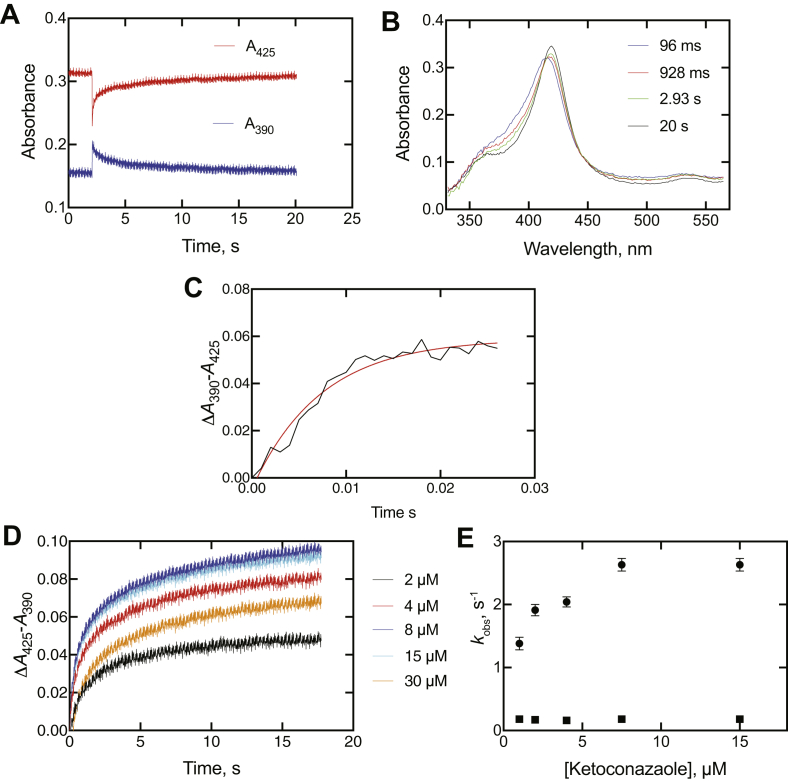
**Binding of ketoconazole to cytochome P450 (P450) 3A4.** The final P450 3A4 concentration was 2 μM, and the final ketoconazole concentration was 15 μM (in 100 mM potassium phosphate buffer, pH 7.4). *A*, spectral traces at 390 and 425 nm. *B*, spectra acquired at the indicated times after mixing, as indicated. The data were collected in the OLIS Show Pre-trigger Mode, with 2.1 s of data from the previous run (completed reaction) shown prior to mixing. *C*, time course of early Δ*A*_390_ − *A*_425_ change in the early phase after mixing. *D*, traces of Δ*A*_425_ − *A*_390_ following the first 18 s after mixing, as a function of ketoconazole concentration. *E*, plots of *k*_obs_ values from biexponential fits of data of part *D* plotted versus final ketoconazole concentration.

Binding of all five inhibitors was qualitatively similar (Figs. 8 and S3–S6). As seen in the preliminary experiments (Fig. 6*C*), the traces at 390 and 425 nm were always in opposite directions (Fig. 8*A*). As pointed out earlier (Fig. 6*C*), data were collected in the Show Pre-trigger Mode, with a short amount of data from the previous run (completed reaction) shown prior to the trace after mixing.

Some individual spectra are shown for ketoconazole binding in Figure 8*B*. An early spectrum (96 ms) after mixing is shown (compare with Fig. 6*E*). It changed to a series of isosbestic spectra, shown with the 928 ms, 2.93 s, and 20 s data. The isosbestic point of these latter spectra (at 411 nm) is at an absorbance value below that in the initial spectrum of the enzyme·intermediate complex (96 ms).

Furthermore, the initial change is not a mixing artifact because of the residual complexed enzyme in the cell, in that the Δ*A*_390_ − *A*_425_ change occurred over the first 25 ms (Fig. 8*C*), an order of magnitude larger than the mixing (“dead”) time (∼2 ms).

The multiphasic increase in absorbance related to the final type II complex (Δ*A*_425_ − *A*_390_) developed over 20 s (and was still not fully complete) (Fig. 8, *A* and *D*). Interestingly, the amplitude of the absorbance change decreased at the highest concentration of ketoconazole (15 μM final).

The results with clotrimazole, ritonavir, indinavir, and itraconazole were all qualitatively similar to those seen with ketoconazole (Figs. S3–S6). In the cases of clotrimazole and ritonavir (Figs. S3*B* and S4*B*), the distinction between the first spectrum (96, 48 ms, respectively) and the subsequent isosbestic spectra is very clear. The Δ*A*_425_ − *A*_390_ data following the formation of the initial spectral complex (Figs. 8*D* and S3*D*–S6*D*) were fit to biexponential plots, and plots of the *k*_obs_ values versus concentration are shown in Figures 8*E* and S3*E*–S6*E*. As in the case of ketoconazole (Fig. 8*D*), the biexponential *k*_obs_ values for binding of the ligands either did not increase or only increased slightly (and in a nonlinear fashion) with the concentration of ligand (Figs. S3*E*–S6*E*).

The *k*_obs_ values of the initial Δ*A*_390_ − *A*_425_ increases (Figs. 8*C* and S3*C*–S6*C*) are compiled in Table 1. Although these may not be true first-order rates, the ratio of rate/ligand concentration gives rough estimates of on-rate constants in the range of 0.9 to 8.1 × 10^7^ M^−1^ s^−1^, consistent with diffusion-limiting binding (15).

### SVD analysis of binding

The initial binding of all inhibitors was very rapid (Table 2) to form the initial blue-shifted complex, *i.e.*, hypsochromic shift (Table 2; Figs. 6*D*, 8*C*, and S3*C*–S6*C*). However, the analysis of the kinetics of the succeeding changes is complex in that the changes are not of first order (Figs. 8*D* and S3*D*–S6*D*), and two wavelengths may not capture the appropriate wavelengths for the transitions (*e.g.*, Fig. 8*A*).

**Table 2:** Initial rates of binding of inhibitory ligands to P450 3A4

Ligand	Concentration, μM^a^	*k*_obs_, s^−1^
Ketoconazole	2	137
Clotrimazole	4	235
Ritonavir	4	650
Indinavir	4	70
Itraconazole	2	195

P450, cytochrome P450.

a Final concentration in flow cell. In each case, the final concentration of P450 3A4 was 2 μM.

An alternative approach to analysis of complex spectral changes is singular value decomposition (SVD) analysis, in which the complete spectra are utilized, not only two individual wavelengths. Use of the OLIS GlobalWorks SVD program indicated that the most appropriate fits to the data involved three intermediate species (not including the ligand-free enzyme), the first of which is the rapidly formed initial E·I complex for which the kinetics are presented in Table 2, not two species. Adding more species is possible but is not necessary (nor justified).

**Figure 9:**
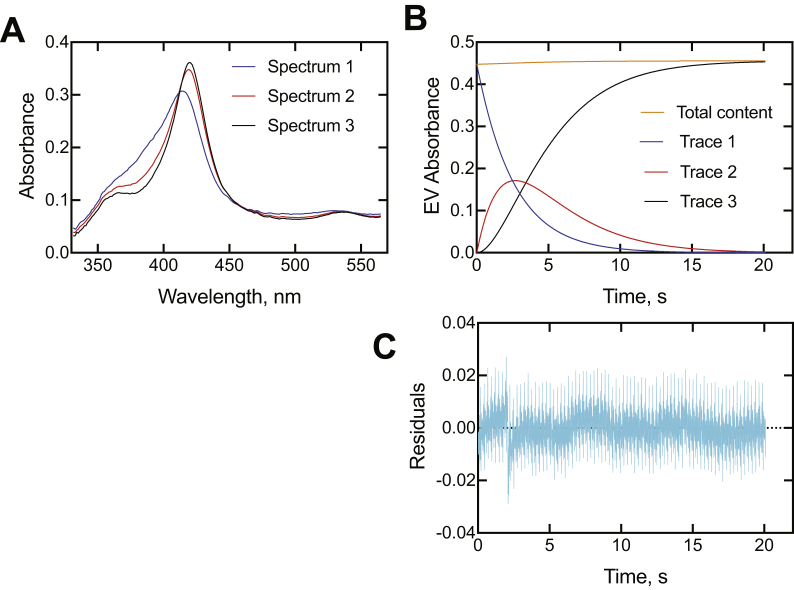
**Singular value decomposition analysis of binding of ketoconazole to cyotochrome P450 (P450) 3A4.** The final concentrations (after mixing) of P450 3A4 and clotrimazole were 2 and 15 μM, respectively. The OLIS GlobalWorks model used was a three-species 1 → 2 → 3 (A → B → C in software) fast/slow rate model, where the unbound P450 3A4 is not included, and 1, 2, and 3 are three different P450 3A4·ketoconazole complexes (this sequence would begin ∼100 ms after mixing P450 3A4 and ketoconazole; Fig. 6, *C*–*D*). *A*, spectra of the three intermediate species (species 1—*blue*; species 2—*red*, and species 3—*black*); *B*, time courses of the three species (same color pattern), plus a plot of the total absorbance accounted for at each time point; *C*, total residual plot for the kinetics traces. The data were collected in the Show Pre-trigger Mode, with 2.1 s of data from the previous run (completed reaction) showing prior to mixing (not reflected in part *B*). See Table 1 for calculated rates.

**Figure 10:**
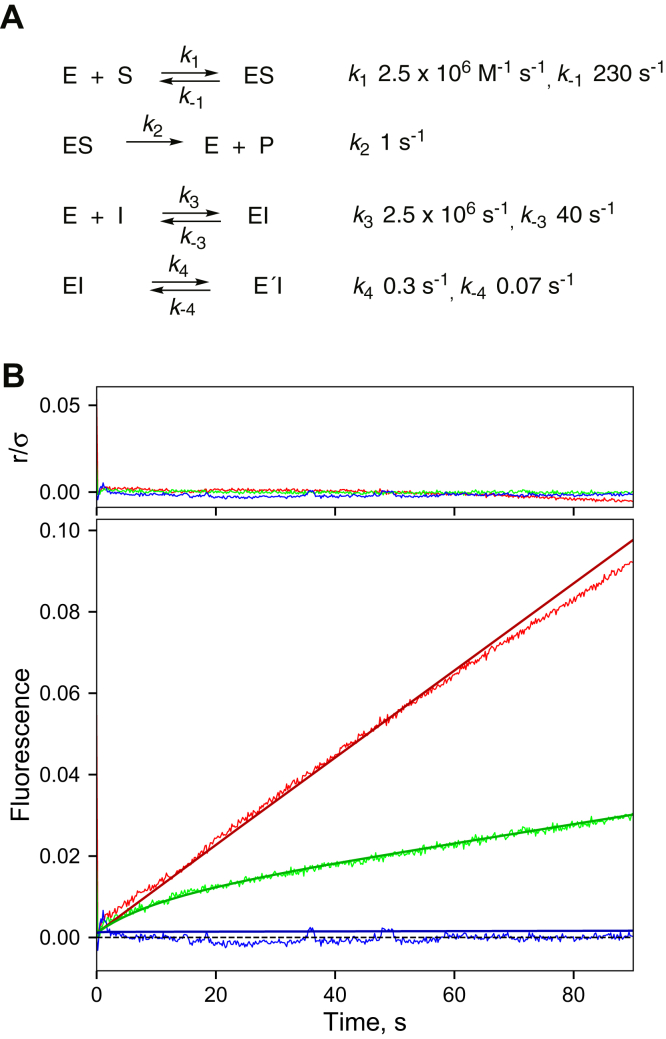
**Analysis of kinetics of inhibition of cytochrome P450 3A4-catalyzed inhibition of 7-benzoyl debenzylation by ketoconazole.***A*, basic model; *B*, ketoconazole (*red*: no inhibitor; *green*: 0.12 μM ketoconazole; and *blue*: 4 μM ketoconazole). The upper section (r/σ) is a residual plot. See similar analyses for ritonavir, indinavir, and itraconazole in Figures S11–S13, with values for *k*_−3_, *k*_4_, and *k*_−4_ adjusted.

SVD analyses of all five P450 3A4–inhibitor associations are shown in Figures 9 and S7–S10. In all cases, the putative intermediate spectra are similar and resemble those reconstructed in Figures 8*B* and S3*B*–S6*B*. The rates of conversion were all in the range of 0.09 to 0.73 s^−1^ and 0.016 to 0.61 s^−1^ for the two reactions, which are the same magnitude as the onset of inhibition values (Fig. 4 and Table 1). The OLIS GlobalWorks model did not account for any reversibility. With regard to other rates and estimates, the values are similar to the slower of the two biexponential rates measured for inhibitor binding to P450 3A4 (Figs. 8*D*–*E*, S3*D*–*E*, S4*D–E*, S5*D–E*, and S6*D–E*) and to the rate constant *k*_4_ in the KinTek Explorer model (Figs. 10*A* and S11–S13).

### Modeling of inhibition data

A minimal model for the onset of 7-OBz quinoline *O*-debenzylation inhibition data (Fig. 4) was developed in KinTek Explorer software. The basic elements are substrate binding, driven by the measured on rate (∼27 s^−1^ at a concentration of 125 μM; Fig. S1*C*) and a *K*_d_ of 90 μM measuring from binding spectra (Fig. S1*B*), a measured *k*_cat_ of ∼1 s^−1^ (Fig. S2), and a rapid binding of the inhibitors to form an initial complex (Figs. 8*C* and S3*C*–S6*C*; Table 1). A reversible conformational change of the EI complex (EI ⇄ E′I) was also included (Figs. 10 and S11–S13).

The data for one of the P450 3A4 inhibitors were not useful for modeling in that the clotrimazole inhibition was too strong (even at a concentration equal to that of the enzyme; Fig. 4*B*). The data for the other four P450 3A4 inhibitors could be fit to a model in which the *K*_d_ for the substrate was fixed at 90 μM (Fig. S1*B*), and the on-rates for both substrate (7-OBz quinoline) and inhibitor were 2.5 × 10^6^ M^−1^ s^−1^, a reasonable value for general binding of ligands to enzymes (15). These values are in agreement with experimental rates, although not as fast as the experiments might suggest (Table 2). (Increasing *k*_1_ in the model did not alter the fits if *k*_−1_ was locked to keep *K*_d_ = *k*_−1_/*k*_1_ = 90 μM; Figs. 10 and S1*B*.)

The fits were driven by adjusting the combination of *k*_−3_, *k*_4_, and *k*_4_ for ketoconazole (Fig. 10). Values for *k*_1_, *k*_−1_, *k*_2_, and *k*_3_ are based on interactions with the substrate 7-OBz quinoline and the initial binding of inhibitor and were held constant for all the inhibitors. The best fits were for some concentrations of ketoconazole (Fig. 10), ritonavir (Fig. S11), and itraconazole (Fig. S13).

If *k*_−4_ was set to zero, a case of mechanism-based inhibition resulted, and the time courses for inhibition (Fig. 4) were exponential, never becoming linear. If only strong inhibition was considered (*i.e.*, *k*_4_ and *k*_−1_ < 0.001 s^−1^), then inhibition occurred immediately with only a linear course of product formation.

## Discussion

**Figure 11:**
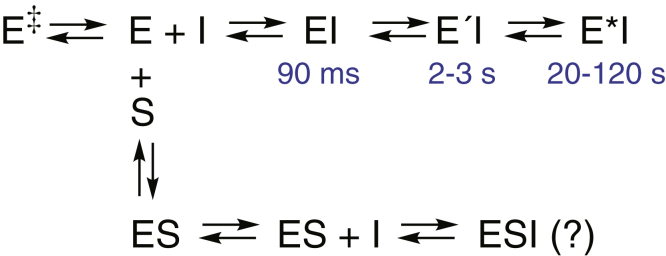
**Scheme summarizing interaction of cytochome P450 3A4 with inhibitors.** The times of appearance of individual species are indicated in *blue*. E, enzyme; I, inhibitor, S, substrate.

Although the literature regarding the inhibition of P450 3A4 (and other P450s) in drug metabolism is considerable (4, 5, 6, 12, 16, 19, 20, 21, 22, 23, 24, 25, 26, 27, 33, 34, 35, 36, 37), there are many discrepancies and issues to resolve. We examined five classic azole and pyridine inhibitors of P450 3A4 (Fig. 1), all of which have clinical relevance. We had shown that the binding of these compounds was slow, as judged by the progression to final complexes (34), and these observations were confirmed by others (36). However, the relevance of the slow kinetics of the steps to enzyme inhibition was unclear. The kinetics were investigated in more detail, and the results are interpreted in the context of a three-step progression to a final complex, which is the most inhibited (Fig. 11).

The initial complex formed after binding each inhibitor clearly shows a blue shift (*i.e.*, hypsochromic, to lower wavelength) (Figs. 6, *E*–*F*, 8*B*, and S3*B*–S6*B*), supported by SVD analyses (Figs. 9*A* and S7*A*–S10*A*). The shift is not to the completely high-spin form (λ_max_ ∼390 nm), devoid of H_2_O as the distal ligand. It could be the result of a partial displacement of H_2_O. However, another viable and perhaps more reasonable proposal is that it represents a structure with an H_2_O molecule sandwiched between the iron atom and a ligand, as clearly demonstrated by X-ray crystallography for binding of (*R*)-bicalutamide to P450 46A1 by Mast *et al*. (29). However, obtaining a crystal structure for such a transient P450 3A4 complex is impractical (and could probably not even be achieved by time-resolved crystallography because of its nature; Fig. 8*B*).

The kinetic modeling (Fig. 10) is oversimplified, for a number of reasons: (i) although there is evidence for a conformational selection model and multiple forms of P450 3A4 in the absence of ligand (38), this equilibrium was not included in the modeling. (ii) Only two EI complexes were included, not the three implicated in SVD analyses, in the attempt to generate a minimal model. (iii) X-ray crystal structures show that multiplicity of ketoconazole binding is at least possible (*i.e.*, two molecules of ketoconazole bound) (11), and thus it is entirely conceivable to have an enzyme–substrate–inhibitor complex, even if one has never been reported. All these possibilities could be considered in a kinetic model, but the rate constants could be indeterminate, and nothing could be proven, even if the fitting were improved.

This work on the binding of inhibitors to P450 3A4 can be considered in the broader context of binding of other ligands, particularly substrates, to P450s. We have examined the binding of substrates to human P450s 3A4, 17A1, and several others and concluded that most involve the dominance of a conformational selection model in which multiple forms of the P450s are in equilibrium and then bind to the substrate (38, 39). However, the results do not preclude the presence of multiple states of substrate-bound P450s, as we have seen with the P450–inhibitor complexes. Thus, there are elements of what can be considered induced fit mechanisms involved here. Induced fit is a term usually used to describe the interactions of enzymes with substrates, but the concept can be applied to the binding of inhibitors or even accessory proteins (as discussed later, all five of the inhibitors studied here; Fig. 1) are not only inhibitors but also substrates. The strong role of conformational selection is evidenced in the lack of increased rates of binding with higher substrate concentrations (Figure 3, Figure 4, Figure 5, Figure 6*E*), except possibly in the case of ketoconazole (Fig. 8*E*). Conformational selection and induced fit are two faces of the importance of protein flexibility in enzyme catalysis but are not necessarily exclusive. Another aspect of the interaction of the inhibitors (Fig. 1) with P450 3A4 is that all these are also substrates for P450 3A4, and P450 3A4 is the major enzyme involved in oxidative metabolism (51, 52, 53, 54) (https://www.accessdata.fda.gov/drugsatfda_docs/label/2013/018533s040lbl.pdf). The literature identifies 2-chlorophenyl-bis-phenylmethane, 2-chlorophenyl,-4-hydroxyphenyl, phenyl methane, and 2-chlorophenyl-bis-phenyl methanol (Fig. S14) as major *in vivo* metabolites of clotrimazole (57), but the enzymes responsible for their formation have not been identified to our knowledge. In order to determine if clotrimazole is a substrate for P450 3A4, as are the other four inhibitors studied here, we incubated clotrimazole with the P450 enzyme system and NADPH and analyzed the products by ultraperformance liquid chromatography (UPLC)-MS (Fig. S15). Two peaks with an apparent MH+ ion at *m*/*z* 295.0884 were formed, corresponding to the loss of the imidazole group and addition of an oxygen. On the basis of the reported metabolism of clotrimazole (57, 58), these are probably 2-chlorophenyl, 4-hydroxyphenyl, phenyl methane, and 2-chlorophenyl-bis-phenyl methanol, although we do not have authentic standards for comparison. Although the literature reports oxidation of imidazole rings (58, 59), the bond to a trisubstituted carbon makes postulation of a mechanism more difficult. One possibility for formation of 2-chlorophenyl-bis-phenyl methanol involves formation of an N-oxide and loss of N-hydroxy (N-OH) imidazole (Fig. S14). The formation of 2-chlorophenyl, 4-hydroxyphenyl, and phenyl methane may be more complex. One possibility is that the imidazole group is lost in a reductive P450 reaction, as with CCl_4_ (60), to form 2-chlorophenyl-bis-phenyl methane, which is then hydroxylated on a phenyl ring to yield 2-chloro, 4-hydroxyphenyl, and phenyl methane (or on the methane carbon to yield 2-chlorophenyl-bis-phenyl methanol). However, no 2-chlorophenyl-bis-phenylmethane was detected in incubations (monitoring *m*/*z* 278).

Although the five inhibitors are different in size and structure, all are small enough (Fig. 1) to go into the active site, based on what is known about its size (∼1400 Å^3^). Four are azoles, and the fifth inhibitor (indinavir) is a pyridine. From all the spectral and structural information available, the final complex (type II) involves Fe–N bonding. Apparently, there are similarities in terms of how these molecules enter the enzyme, shift the heme environment to yield initial spectral changes, and then settle in to form the final complexes. All must be in some equilibrium to be oxidized by the enzyme as well.

In the absence of complete identification of the clotrimazole products, these experiments do not establish a mechanism but do establish that (i) as with the other four inhibitors (Fig. 1), clotrimazole is a substrate as well as an inhibitor of P450 3A4 and (ii) that some alternate enzyme/ligand/heme positioning is required for product formation compared with inhibition. As pointed out by Pearson *et al*. (37), an alternate conformation of P450 3A4 bound to the azoles should be required for oxidation as opposed to inhibition, in that tight binding of a nitrogen atom precludes binding of O_2_ (to ferrous P450). In the scheme in Figure 11, the final E ∗ I complex should not be a substrate but EI or E′I might be. Alternatively, as proposed by Pearson *et al*. (37), there might be a parallel pathway leading to a catalytically competent complex.

Even among extensively studied model of bacterial P450s, the dynamics of ligand binding is not simple. P450_BM-3_ (CYP102A1) appears to utilize a conformational selection model in binding both the substrates dodecyl sulfate and myristate (61). P450_cam_ (CYP101A1) binds its substrate camphor in a rapid reaction, leading to high-spin iron, in a kinetic mechanism that can be described by a two-state model with rapid binding (62). However, various biophysical approaches have been employed to demonstrate the existence of multiple conformational states of ligand-bound P450_cam_ (60, 62, 63). The binding of camphor can be understood in the context of a mechanism dominated by induced fit, although alternative substrates for the enzyme do not seem to fit this paradigm (66). A related P450 from a (bacterial) pseudomonad, P450_tcu_, catalyzes the same 5-*exo* hydroxylation of camphor with nearly equal efficiency, but the P450_tcu_ transition to high-spin iron was characterized by biphasic kinetics, with 40% being rapid (rate not determined) and another 30% with a long *t*_1/2_ of ∼25 min (67). Exactly how the slow step relates to catalysis (which is ∼25,000 times faster than the spin state conversion) is unclear.

Surface plasmon resonance methods have been applied to study P450 3A4 interactions with itraconazole and ketoconazole (37). That method involves immobilization of the P450 on a chip, and a major deficiency of the approach is mass transfer, a term used to describe the diffusion of the ligand from the solution through the matrix to reach the receptor (15). The surface plasmon resonance model described by Pearson *et al*. (37) had two on-rates, which does not have an obviously relevant physical meaning in terms of enzyme kinetics. Alternatively, there is an initial diffusion-limited encounter and then rate constants to characterize the subsequent steps. Furthermore, as in the case of many surface plasmon resonance studies (15), the rate constants are too slow to be near to being diffusion limited (∼10^4^ M^−1^, s^−1^; compare with Table 1).

Chuo *et al*. (68) have characterized the structures of P450 3A4 bound to ketoconazole and ritonavir, utilizing double electron–electron resonance, molecular dynamics, and previous crystal structures, and concluded that the average global structures of P450 3A4 did not undergo major changes upon binding of these inhibitors. However, the binding of the substrate midazolam resulted in major changes in motion and/or disorder in the F/G helix region near the substrate-binding pocket. The relationship of the structural work (68) with the present kinetic studies is unclear. The structural studies involve the final stable complex (Fig. 11), but the structures of intermediate forms cannot be determined without the use of time-resolved crystallography.

One question that our results raise is exactly when does P450 3A4 inhibition begin, if there are multiple states following binding of the inhibitors (Fig. 11). The most relevant guide is probably the results presented in Figure 4. In all cases except that of itraconazole, full inhibition was not seen (at the lower concentration) for several seconds. This result seems to indicate that the initial complex (EI in Fig. 11) is not very inhibitory and that the E′I and E ∗ I complexes are. If the initial EI complex involves placement of the inhibitor in the substrate-binding site, then one would expect immediate inhibition. We do not know the off-rate of the substrate, but using a *k*_obs_ value of 27 s^−1^ at 62 μM (Fig. S1*C*), the on-rate constant should be >5 × 10^5^ M^−1^ s^−1^. With a *K*_d_ of 90 μM, an off-rate of 45 s^−1^ can be calculated for a simple two-state system and a *t*_1/2_ of 15 ms. These calculations suggest that the substrate is not being displaced. Given the size of the active site (≥1400 Å^3^) (9, 11) and the sizes of the inhibitors (Fig. 1) and substrates (∼325 Å^3^ for 7-OBz quinoline, ∼380 Å^3^ for testosterone), it is possible that the inhibitor and substrate can both occupy the active-site area in the initial complex. Conversion to the final complex (E ∗ I in Fig. 11) leads to a new steady state and more complete inhibition.

In summary, the binding of at least some inhibitors to P450 3A4 is multiphasic. This phenomenon may have relevance to the time-dependent inhibition often experienced with the enzyme in pharmaceutical development programs (6, 20). Whether the phenomena seen here fit into what is classically termed slow and tight-binding inhibition (15, 46, 69) will require more investigation. The results, coupled with earlier studies on conformational selection (38) and the considerable structural evidence for multiple conformations of P450 3A4 (9, 11), support a very dynamic picture of this enzyme, which is consistent with its broad catalytic specificity. However, there are many elements of regioselectivity and stereoselectivity associated with P450 3A4 (70), and the catalytic courses that the enzyme have some boundaries.

## Experimental procedures

### Chemicals

Indinavir was purchased from USP. The other four inhibitors were from Sigma–Aldrich. All were used without further purification.

### Synthesis of 7-OBz quinoline

7-OH quinoline (Acros Organics [Thermo Fisher]) was heated with benzyl bromide in dimethylformamide (with K_2_CO_3_) to prepare 7-OBz quinoline as described elsewhere (70, 71). The product was purified by column silicic acid chromatography (gradient of increasing ethyl acetate in hexanes, eluting with 50% ethyl acetate) in 33% yield. The product was further purified by preparative TLC (silica gel G, 2 mm, CHCl_3_–acetone, 19–1, v/v). MS *m/z* 236.2 (MH^+^), NMR (400 MHz, CDCl_3_) *δ* 5.24 (2H, s, –CH_2_–), 7.3 to 7.5 (aromatic), 7.81 (1H, d, H-5), 8.42 (1H, d, H-4), 8.51 (1H, d, H-2); mp 225 to 228 °C (dec), lit. 72.2 to 73.2 °C (71); UV (C_2_H_5_OH) ε_232_ 35,700 M^−1^ cm^−1^, and ε_332_ 4650 M^−1^ cm^−1^. The mp does not match the only one reported in the literature (71), but the mode of synthesis and the spectral and other properties leave no doubt that this is the specified product.

### Enzymes

P450 3A4, with a modified N-terminal (72) and C-terminal (His)_6_ tag (43), was expressed in *Escherichia coli* and purified as described (43, 73). Rat NADPH–P450 reductase (POR) and human cytochrome *b*_5_ were expressed in *E. coli* and purified as described (74, 75).

For catalytic assays, P450 3A4 was reconstituted with a cholate/phospholipid mixture as described previously (42, 43). In general, reactions were initiated either with NADPH (1 mM) or an NADPH-generating system (76).

### 7-OBz quinoline inhibition assays

Assays for the kinetics of inhibition were done using an OLIS RSM-1000 stopped-flow instrument (On-Line Instrument Systems) in the fluorescence mode (150 W xenon lamp), with the excitation monochromator set at 410 nm and using a >515 nm Oriel long-pass filter (Newport) with the sample photomultiplier tube. The slit widths were 6.32 mm, and the sample and reference voltages of the photomultiplier tubes were 700 and 350 V, respectively. Reactions were done at 23 °C for 180 s.

One syringe contained the P450 3A4 system (100 nM P450 3A4, 200 nM POR, and 100 nM cytochrome *b*_5_) along with the other components of a “5×” mixture (potassium Hepes (pH 7.4), reduced glutathione, sodium cholate, and phospholipid mixture) combined with the 5× buffer mixture (Hepes, MgCl_2_, and reduced glutathione) (42), plus NADPH and the inhibitor compound. Fluorescence data were collected for 3 min (at 23 °C) and moved to Excel, Prism, and KinTek Explorer programs for analysis.

In the IC_50_ determinations, 100 μl reaction mixtures were prepared by mixing equal volumes of the 5× P450 3A4 reconstitution mix and the 5× buffer mix on ice. Water was added, and 7-OBz quinoline (in ethanol) was aliquoted at 0.5% (v/v) to a final concentration of 20 μM. Inhibitors (in dimethyl sulfoxide) were added (final concentration 0.5% dimethyl sulfoxide, v/v) for their desired final concentrations (0–10 μM), bringing the total organic solvent composition of the reaction to 1% (v/v). Preincubations proceeded at 37 °C for 5 min and were initiated with the addition of the NADPH-generating system (76). Reactions (8 min) were quenched with 100 μl of cold CH_3_CN on ice and centrifuged at 2000*g* for 10 min to pellet precipitated protein. Aliquots of samples (200 μl) were transferred to vials, and the product (7-OH quinoline) was measured by UPLC using a 2.1 × 100 mm Acquity BEH octadecylsilane (C18) column (1.7 μm) (25 °C) with a Waters Acquity UPLC instrument. Samples (held at 4 °C) were injected (10 μl) with a flow rate of 0.2 ml min^−1^ using a CH_3_CN/H_2_O (20 mM NH_4_CH_3_CO_2_) gradient as follows (44): 0 min, 15% CH_3_CN; 0.1 min, 15% CH_3_CN; 6 min, 40% CH_3_CN; 10 min, 90% CH_3_CN; 11 min, 90% CH_3_CN; 11.1 min, 15% CH_3_CN; 12 min, 15% CH_3_CN (all v/v). The product 7-OH quinoline was detected using a Waters Acuity fluorescence detector with excitation and emission wavelengths set at 420 and 503 nm, respectively. Data were processed using the MassLynx software, and the velocity of product formation (picomole [pmol] formed per minute per pmol of P450 3A4) was calculated by comparison to a 7-OH quinoline standard curve. IC_50_ fits for inhibition were made using GraphPad Prism (La Jolla, CA) and the following equation: *Y* = bottom + (top–bottom)/ + 1+10^((*X*-LogIC50))^, where *Y* = response, *X* = log(dose), top/bottom = plateaus in same units as *Y*.

### 7-OBz quinoline rescue assays

An enzyme mixture, as used in the 7-OBz quinoline inhibition assays (50 nM P450 3A4, 100 nM POR, and 50 nM cytochrome *b*_5_), was mixed with 50 nM inhibitor and held at room temperature for 20 min. An NADPH-generating system (76) was added, and the reaction (volume 2.5 ml) was initiated in an OLIS DM-45 fluorimeter by the addition of 160 μM 7-OBz quinoline (in C_2_H_5_OH). The slit widths were both 6.32 mm, respectively, and the excitation and emission wavelengths were 410 and 510 nm, respectively. Assays were done at 23 °C, and the fluorescence of the product 7-OH quinoline was measured in 10 × 10 mm cells mirrored on two sides to increase sensitivity (Starna Cells; catalog 23-Q-10-MC).

Incubations proceeded for 15 min, and the data files were processed in Excel, Prism, and KinTek Explorer.

### Testosterone 6β-hydroxylation inhibition assays

The same reconstituted P450 3A4 system was used as for the 7-OBz quinoline assays, and the experiments followed largely the same procedure with some exceptions: incubations were done for the IC_50_ determinations using 25 μM testosterone (15 min at 37 °C), and the product (6β-OH testosterone) was measured by UPLC using a 2.1 × 100 mm (1.7 μm) Acquity BEH octadecylsilane (C_18_) column (25 °C) with a Waters Acquity UPLC instrument. Samples (held at 4 °C) were injected (10 μl) with a flow rate of 0.4 ml min^−1^ using a CH_3_OH gradient as follows (77): 0 min, 5% CH_3_OH; 0.1 min, 5% CH_3_OH; 2.5 min, 95% CH_3_OH; 4 min, 95% CH_3_OH; 4.1 min, 5% CH_3_OH; and 5 min, 5% CH_3_OH (all v/v). The product 6β-OH testosterone was detected at 245 nm. Data were processed using the MassLynx software, and the velocity (pmol product formed per minute per pmol P450 3A4) was calculated by comparison to a 6β-OH testosterone standard curve. IC_50_ fits for inhibition were made using GraphPad Prism as in the case of 7-OH quinoline (see aforementioned).

The kinetic inhibition assays (Fig. 5) were done with the same basic reconstituted system, but the enzyme concentrations were increased tenfold (5 μM P450 3A4, 10 μM POR, and 5 μM cytochrome *b*_5_) for the indicated times at 37 °C using a KinTek RQF-3 rapid quench apparatus (KinTek). Reactions were initiated by mixing 19 μl of the enzyme system (with 20 μM [1,2,6,7-^3^H]-testosterone, 0.5 Ci/mmol) in one side and 19 μl of NADPH (10 mM) plus the indicated amount of inhibitor in the other side. The reaction started with NADPH addition, and any lag for inhibition would be observed. After the indicated amount of time, each reaction was stopped by the addition of 160 μl of 1 M HCl, giving a fivefold dilution of the reaction mix to 200 μl. The products of five separate reactions (from each time point) were combined, and the products and substrate were extracted into 2 ml of ethyl acetate. An aliquot (1.5 ml) of the organic (upper) layer was transferred to a new vial, the solvent was removed under a stream of N_2_ gas, and the residue was dissolved in 125 μl of 50% aqueous CH_3_CN (v/v). An aliquot of each sample (held at 4 °C) was injected onto an Agilent 1100 HPLC instrument using a Beckman Ultrasphere 4.6 × 250 mm octadecylsilane column (C_18_) (5 μm) with a gradient of an aqueous 0.1% HCO_2_H solution (w/v) and CH_3_CN at a flow rate of 1 ml min^−1^ as follows: 0 min, 60% CH_3_CN; 4 min, 60% CH_3_CN; 10 min, 20% CH_3_CN; 11 min, 20% CH_3_CN; 12 min, 60% CH_3_CN; and 14 min, 60% CH_3_CN (all v/v). Product was detected by mixing the effluent from the column with a stream of scintillation cocktail (2 ml min^−1^) and detection of the radioactivity with a β-RAM model 5 system (IN/US; LabLogic). The dpm in the 6β-OH testosterone peaks were converted to pmol product formed, using the percentage values for the substrate and product peaks for correction.

### Substrate-binding titrations

P450 3A4 (2.0 μM) was dissolved in 100 mM potassium phosphate buffer (pH 7.4). Two 1.0 ml cuvettes were used in an OLIS-Cary14 or OLIS-Aminco DW-2 spectrophotometer, and a baseline was established (23 °C). Increasing microliter amounts of 7-OBz quinoline (Fig. S1) or ketoconazole (Fig. 6*A*) were added to the sample cuvette using a cuvette mixer (Bel-Art) (from ethanolic solutions). Spectra were recorded, and the difference Δ*A*_390_ − *A*_420_ at each concentration was used to estimate *K*_d_ for 7-OBz quinoline with a hyperbolic fitting equation in GraphPad Prism software, with the enzyme concentration E fixed.

### Inhibitor-binding kinetics

All measurements were made at 23 °C in 20 mM potassium phosphate buffer (pH 7.4), using an OLIS RSM-1000 stopped-flow spectrophotometer equipped with a 16 × 0.2 mm spinning disk, acquiring 4000 scans over 4 s or, for a period of 20 to 120 s, averaging 62 scan s^−1^. The slits were both 1.24 mm and 400 lines/mm, greater than 500 nm gratings were used, covering a 332 to 565 nm wavelength range. Equal volumes of buffered solutions of P450 3A4 and ligand were mixed, with a nominal dead time of 2 ms. Data were collected in the Show Pre-trigger Mode, with a short amount of data from the previous run (completed reaction) shown prior to mixing. This approach is useful in that the expected endpoint is displayed and provides an estimate of whether the reaction has gone to completion in the time frame it is being monitored. From the accumulated spectra from each individual experiment (5–10 shots per concentration), generally the *A*_420_ data were subtracted from the *A*_390_ data points or *A*_390_ data were subtracted from the *A*_425_ data points, and the difference (Δ*A*_390_ − *A*_420_ or Δ*A*_425_ − *A*_390_ versus time) traces were averaged using the OLIS GlobalWorks software and fit to first-order fits (or used in SVD analysis; Figs. 9 and S7–S10). The Δ*A*_390_ − *A*_420_ and Δ*A*_425_ − *A*_390_ data sets were saved as Excel files, transferred to an Apple Mac OS 10.15.6 system, saved as txt files, and in some cases used in GraphPad Prism or KinTek Explorer software.

## Data availability

All data are contained within the article and the supporting information.

## Conflict of interest

The authors declare that they have no conflict of interest with the contents of this article.
