# Supplementary material for: Kinetics of cytochrome P450 3A4 inhibition by heterocyclic drugs defines a general sequential multistep binding process
Source: J Biol Chem. 2020 Dec 25;296:100223. doi: 10.1074/jbc.RA120.016855 (PMC7948456; doi:10.1074/jbc.RA120.016855)
Supplement: Figures S1 to S15 [file mmc1.docx]

**Supporting Information**

**Kinetics of cytochrome P450 3A4 inhibition by heterocyclic drugs defines a general sequential multi-step binding process**

F. Peter Guengerich, Kevin D. McCarty, and Jesse G. Chapman

Table of Contents

Figure S1. Binding of 7-OBz quinidine to P450 3A4.

Figure S2. P450 3A4-catalzyed 7-OBz quinidine *O*-debenzylation activity as a function of substrate concentration.

Figure S3. Binding of clotrimazole to P450 3A4.

Figure S4. Binding of ritonavir to P450 3A4.

Figure S5. Binding of indinavir to P450 3A4.

Figure S6. Binding of itraconazole to P450 3A4.

Figure S7. SVD analysis of binding of clotrimazole to P450 3A4.

Figure S8. SVD analysis of binding of ritonavir to P450 3A4.

Figure S9. SVD analysis of binding of indinavir to P450 3A4.

Figure S10. SVD analysis of binding of itraconazole to P450 3A4.

Figure S11. Analysis of kinetics of inhibition of P450 3A4-catalyzed inhibition of 7-OBz debenzylation by ritonavir.

Figure S12. Analysis of kinetics of inhibition of P450 3A4-catalyzed inhibition of 7-OBz debenzylation by indinavir.

Figure S13. Analysis of kinetics of inhibition of P450 3A4-catalyzed inhibition of 7-OBz debenzylation by itraconazole.

Figure S14. Potential pathways for formation of clotrimazole products.

Figure S15. Formation of clotrimazole products by P450 3A4.

**Figure S1. Binding of 7-OBz quinidine to P450 3A4.** *A*, steady-state binding. *B*, estimation of *K*_d_. The concentration of P450 3A4 was 2 µM in Parts *A* and *B*, and the *K*_d_ was 90 µM (± 21 µM). *C*, pre-steady-state binding kinetics. One syringe contained 4 µM P450 3A4 in 100 mM potassium phosphate buffer (pH 7.4). The second syringe contained 125 µM 7-OBz quinoline in 100 mM potassium phosphate buffer (pH 7.4). The two solutions were mixed in an OLIS RSM-1000 stopped-flow spectrophotometer set up in the rapid scanning mode with 1.24 mm slits and 400 L/mm, 500 nm> gratings (23 °C). The averaged Δ*A*_390_-*A*_418_ traces are shown for a reaction of 4 s. No further change was observed up to 20 s (data not shown). The (single) exponential *k*_obs_ was 27 ± 1 s^-1^ (black line).

**Figure S2. P450 3A4-catalzyed 7-OBz quinidine *O*-debenzylation.** *A*, reaction scheme. *B*, activity as a function of substrate concentration. Activity was measured in an OLIS DM45 instrument at 23 °C. The *k*_cat_ was 58 min^-1^, as calculated following the addition of standard amounts of the product 7-OH quinoline (*K*_m_ 21 ± 3 µM).

**Figure S3. Binding of clotrimazole to P450 3A4.** *A*, spectral traces at 390 and 425 nm. The final P450 3A4 concentration was 2 µM and the final clotrimazole concentration was 15 µM (in 100 mM potassium phosphate buffer, pH 7.4). The data were collected in the Show Pre-trigger Mode, with 2.1 s of data from the previous run (completed reaction) showing prior to mixing. *B*, Spectra acquired at the times of 96 ms, 3 s, 18 s, and 118 s after mixing, as indicated. *C*, time course of Δ*A*_390_-*A*_425_ change in early phase after mixing (235 s^-1^). *D*, traces of Δ*A*_425_-*A*_390_ after the first 120 ms after mixing, as a function of clotrimazole concentration. *E*, plots of *k*_obs_ values from biexponential fits of data of Part *D* plotted *vs.* final ketoconazole concentration.

**Figure S4. Binding of ritonavir to P450 3A4.** *A*, spectral traces at 390 and 425 nm. The final P450 3A4 concentration was 2 µM and the final ritonavir concentration was 15 µM (in 100 mM potassium phosphate buffer, pH 7.4). The data were collected in the Show Pre-trigger Mode, with 2.1 s of data from the previous run (completed reaction) showing prior to mixing. *B*, spectra acquired at the times of 48 ms, 0.94 s, 5.9 s, and 18 s after mixing, as indicated. *C*, time course of Δ*A*_390_-*A*_425_ change in early phase after mixing (650 s^-1^). *D*, traces of Δ*A*_425_-*A*_390_ after the first 120 ms after mixing, as a function of ketoconazole concentration. *E*, plots of *k*_obs_ values from biexponential fits of data of Part *D* plotted *vs.* final ketoconazole concentration.

**Figure S5.** **Binding of indinavir to P450 3A4.** *A*, spectral traces at 390 and 425 nm. The final P450 3A4 concentration was 2 µM and the final indinavir concentration was 15 µM (in 100 mM potassium phosphate buffer, pH 7.4). The data were collected in the Show Pre-trigger Mode, with 2.1 s of data from the previous run (completed reaction) showing prior to mixing. *B*, spectra acquired at the times of 96 ms, 2.94 s, 17.9 s, and 118 s after mixing, as indicated. *C*, time course of Δ*A*_390_-*A*_425_ change in early phase after mixing (70 s^-1^). *D*, traces of Δ*A*_425_-*A*_390_ following the first 120 ms after mixing, as a function of indinavir concentration. *E*, plots of *k*_obs_ values from biexponential fits of data of Part *D* plotted *vs.* final ketoconazole concentration.

**Figure S6.** **Binding of itraconazole to P450 3A4.** *A*, spectral traces at 390 and 425 nm. The final P450 3A4 concentration was 2 µM and the final itraconazole concentration was 15 µM (in 100 mM potassium phosphate buffer, pH 7.4). The data were collected in the Show Pre-trigger Mode, with 2.1 s of data from the previous run (completed reaction) showing prior to mixing. *B*, spectra acquired at the times of 32 ms, 928 ms, 2.9 s, and 28 s after mixing, as indicated. *C*, time course of Δ*A*_390_-*A*_425_ change in early phase after mixing (195 s^-1^). *D*, traces of Δ*A*_425_-*A*_390_ following the first 120 ms after mixing, as a function of itraconazole concentration. *E*, plots of *k*_obs_ values from biexponential fits of data of Part *D* plotted *vs.* final ketoconazole concentration.

**Figure S7. SVD analysis of binding of clotrimazole to P450 3A4.** The final concentrations (after mixing) of P450 3A4 and clotrimazole were 2 and 15 µM, respectively. An OLIS GlobalWorks model used was a 3-species A🡒B🡒C fast/slow rate model. *A*, spectra of the three species (species 1—blue; species 2—red, species 3—black); *B*, time courses of the three species (same color pattern), plus a plot of the total absorbance accounted for at each time point; *C*, residuals plot for the kinetics traces. See Table 1 for calculated rates.

**Figure S8. SVD analysis of binding of ritonavir to P450 3A4.** The final concentrations (after mixing) of P450 3A4 and clotrimazole were 2 and 15 µM, respectively. An OLIS GlobalWorks model used was a 3-species A🡒B🡒C fast/slow rate model. *A*, spectra of the three species (species 1—blue; species 2—red, species 3—black); *B*, time courses of the three species (same color pattern), plus a plot of the total absorbance accounted for at each time point; *C*, residuals plot for the kinetics traces. See Table 1 for calculated rates.

**Figure S9. SVD analysis of binding of indinavir to P450 3A4.** The final concentrations (after mixing) of P450 3A4 and clotrimazole were 2 and 15 µM, respectively. The OLIS GlobalWorks model used was a 3-species A🡒B🡒C fast/slow rate model. *A*, spectra of the three species (species 1—blue; species 2—red, species 3—black); *B*, time courses of the three species (same color pattern), plus a plot of the total absorbance accounted for at each time point; *C*, residuals plot for the kinetics traces. See Table 1 for calculated rates.

**Figure S10. SVD analysis of binding of itraconazole to P450 3A4.** The final concentrations (after mixing) of P450 3A4 and clotrimazole were 2 and 15 µM, respectively. An OLIS GlobalWorks model used was a 3-species A🡒B🡒C fast/slow rate model. *A*, spectra of the three species (species 1—blue; species 2—red, species 3—black); *B*, time courses of the three species (same color pattern), plus a plot of the total absorbance accounted for at each time point; *C*, residuals plot for the kinetics traces. See Table 1 for calculated rates.

**Figure S11. Analysis of kinetics of inhibition of P450 3A4-catalyzed inhibition of 7-OBz debenzylation by ritonavir.** See Fig. 10 for basic model used in KinTek Explorer, with *k*_1_, *k*_-1_, *k*_2_, and *k*_3_ (based on interactions with the substrate 7-OBz quinoline and the the initial binding of inhibitor) as in that case and *k*_-3_ = 2.5 s^-1^, *k*_4_ = 0.15 s^-1^, and *k*_-4_ = 0.015 s^-1^. Compare to Fig. 4*C*. Red line: no inhibitor; green line: 0.2 µM ritonavir; blue line: 0.6 µM ritonavir. The solid lines are the fits to the model for the fluorescence, and a residuals analysis of the fits (r/σ) is shown at the top.

**Figure S12. Analysis of kinetics of inhibition of P450 3A4-catalyzed inhibition of 7-OBz debenzylation by indinavir.** See Fig. 10 for basic model used in KinTek Explorer, with *k*_1_, *k*_-1_, *k*_2_, and *k*_3_ (based on interactions with the substrate 7-OBz quinoline and the the initial binding of inhibitor) as in that case and *k*_-3_ = 125 s^-1^, *k*_4_ = 0.1 s^-1^, and *k*_-4_ = 0.01 s^-1^. Compare to Fig. 4*D*. Red line: no inhibitor; green line: 2 µM indinavir; blue line: 6 µM indinavir. The solid lines are the fits to the model for the fluorescence, and a residuals analysis of the fits (r/σ) is shown at the top.

**Figure S13. Analysis of kinetics of inhibition of P450 3A4-catalyzed inhibition of 7-OBz debenzylation by itraconazole.** See Fig. 10 for basic model used in KinTek Explorer, with *k*_1_, *k*_-1_, *k*_2_, and *k*_3_ (based on interactions with the substrate 7-OBz quinoline and the the initial binding of inhibitor) as in that case and *k*_-3_ = 40 s^-1^, *k*_4_ = 0.3 s^-1^, and *k*_-4_ = 0.07s^-1^. Compare to Fig. 4*E*. Red line: no inhibitor; green line: 0.2 µM itraconzole; blue line: 0.6 µM itraconazole. The solid lines are the fits to the model for the fluorescence, and a residuals analysis of the fits (r/σ) is shown at the top.

**Figure S14. Potential pathways for formation of clotrimazole products.** *A*, possibility for formation of 2-chlorophenyl-*bis*-phenyl methanol. *B*, possibility for formation of 2-chlorophenyl-*bis*-phenyl methane, 2-chlorophenyl-*bis*-phenyl methanol, and 2-chlorophenyl, 4-hydroxyphenyl, phenyl methane.

**Figure S15. Formation of clotrimazole products by P450 3A4.** The standard P450 3A4 reaction mixture was incubated with 50 µM clotrimazole for 30 minutes at 37 °C and the products were extracted into two volumes of ethyl acetate, concentrated under a nitrogen stream, and analyzed. The UPLC system used a 2.1 mm × 50 mm Acquity BEH octadecylsilane (C_18_) column (1.7 µm) held at 30 °C and a gradient of (solvent A) 0.1% aqueous HCO_2_H and (B) 0.1% HCO_2_H in CH_3_OH as follows: 0 min, 75% A; 0.5 min, 75% A; 8 min, 0% A; 9 min, 0% A; 9.1 min, 75% A; 12 min, 75% A (all v/v). The effluent was directed into a Thermo Q Exactive HF LC/MS system using an electrospray ionization source operating in the positive ionization mode with a collision energy of 35 eV. Full mass spectrometry analysis was performed between *m/z* 50 and 400, and Parallel Reaction Monitoring (PRM) was used to identify the metabolites. Data were processed using the Thermo Fisher Xcalibur software. *A*, total ion current, showing the residual clotrimazole at *t*_R_ 5. 99 min. *B*, incubation done in the presence of an NADPH-generating system with selected ion monitoring was at *m/z* 295.0884, the exact mass for the protonated forms of both 2-chlorophenyl-*bis*-phenyl methanol and 2-chlorophenyl, 4-hydroxyphenyl, phenyl methane. *C*, same reaction as in Part *B* but done in the absence of NADPH. The conclusion is that the peaks eluted at 5.46 and 5.73 min in Part *B* are probably 2-chlorophenyl-*bis*-phenyl methanol and 2-chlorophenyl, 4-hydroxyphenyl, phenyl methane, based on the *m/z* and literature precedent for metabolites (ref.,59 of body of paper), although we cannot discern which is which from the fragmentation patterns.
